# Pet ownership-related differences in medical and long-term care costs among community-dwelling older Japanese

**DOI:** 10.1371/journal.pone.0277049

**Published:** 2023-01-27

**Authors:** Yu Taniguchi, Yuri Yokoyama, Tomoko Ikeuchi, Seigo Mitsutake, Hiroshi Murayama, Takumi Abe, Satoshi Seino, Hidenori Amano, Mariko Nishi, Yasuhiro Hagiwara, Shoji Shinkai, Akihiko Kitamura, Yoshinori Fujiwara

**Affiliations:** 1 Japan Environment and Children’s Study Programme Office, National Institute for Environmental Studies, Tsukuba, Japan; 2 Research Team for Social Participation and Community Health, Tokyo Metropolitan Institute of Gerontology, Tokyo, Japan; 3 Research Team for Human Care, Tokyo Metropolitan Institute of Gerontology, Tokyo, Japan; 4 Department of Biostatistics, School of Public Health, Graduate School of Medicine, The University of Tokyo, Tokyo, Japan; 5 Faculty of Nutrition, Kagawa Nutrition University, Saitama, Japan; Ehime University Graduate School of Medicine, JAPAN

## Abstract

This study examined the differences in medical and long-term care costs over 18 months between pet owners and non-owners among community-dwelling older Japanese. Pet ownership data were collected from 460 community-dwelling adults age 65 years and older. These data were matched with data from the National Health Insurance, health insurance for older people, and Long-Term Care Insurance beneficiaries for 17 months back from the survey on pet ownership. Pet-ownership group-specific trajectories in monthly medical and long-term care costs were modeled by a generalized estimating equation. Among pet owners (n = 96, 20.9%) and non-pet owners (n = 364, 79.1%) there were no significant differences in baseline demographic or health characteristics including chronic disease and self-reported long-term care level. At baseline, pet owners had estimated monthly medical costs of ¥48,054 (SE = 0.11; $418), compared to ¥42,260 (SE = 0.06; $367) for non-pet owners. The monthly medical costs did not differ significantly between the two groups during the 18-month follow-up period. At baseline, estimated monthly long-term care costs of pet owners and non-pet owners were ¥676 (SE = 0.75; $6) and ¥1,420 (SE = 0.52; $12), respectively. During the follow-up period, the non-pet owner to owner ratio of monthly long-term care costs was 1.2 at minimum and 2.3 at maximum. This study showed that monthly long-term care costs for pet owners were approximately half those of non-pet owners. Pet owners might use long-term care services less frequently, or use lighter care services.

## Introduction

Human–animal interaction (HAI) is the term given for the potentially mutually beneficial relationships that arise between people and animals. Accumulating evidence indicates the psychological, physiological, and social benefits of HAI [1]. People with HAI have higher physical activity levels [1, 2], better mental health [3], and lower social isolation [1, 4] compared to people without HAI. Longitudinal studies have shown that the effects of HAI on adults and older adults include enhanced ordinary activities of daily living (ADL) [5], lower risk of cardiovascular disease [6] and mortality [7], and increased survival rates [8].

Frailty, characterized as a state of physiological vulnerability, has recently attracted increasing research in gerontology. Frailty is reported to be a risk factor for hospitalization [9], disability [10–13], dementia [14], and mortality [15]. Our previous longitudinal studies revealed that the odds ratios for incidence of frailty in community-dwelling adults aged 65 years or older were 0.87 (95% confidence interval [CI]: 0.69–1.09) for current dog or cat owners and 0.84 (0.71–0.98) for past owners, after controlling for important confounders, compared with “never” owners [16, 17]. We also recently reported that older adults who currently owned a dog had a lower odds ratio of onset of disability (OR = 0.54, 95% CI: 0.37–0.79) compared to a reference group that had never owned a dog [18].

This accumulating evidence on HAI suggests that exposure to pets has beneficial effects on the health of their owners. We hypothesized that pet owners consistently use medical and long-term care services less frequently than non-owners throughout the period of ownership, resulting in smaller increases in healthcare costs. To our knowledge, no previous studies have investigated the relationship between pet ownership and medical and long-term care costs.

This study used epidemiological data to analyze the relationship between pet ownership among community-dwelling adults and National Health Insurance, health insurance for older people and Long-Term Care Insurance (LTCI) costs under a universal health insurance system. We compared the medical and long-term care costs over an 18-month period between pet owners and non-owners among community-dwelling older Japanese people.

## Materials and methods

### Participants

Data were collected as part of the Hatoyama Cohort Study, which was launched in 2010. The details of the study design are reported elsewhere [19]. Briefly, the Hatoyama Cohort Study was a prospective cohort study of community-dwelling adults aged 65 years or older living in the town of Hatoyama in Saitama Prefecture, Japan. The study sample was constructed using stratified random sampling, classified by age and residential area. Surveys were conducted every 2 years in the same manner.

Of the 742 participants, 463 residents completed a follow-up survey with questions on pet ownership in June 2017. In this study, we assessed the survey in June 2017 as baseline survey To be eligible for the study, individuals had to complete a questionnaire on their experience of dog or cat ownership. Valid data were received from 460 participants. All participants provided written informed consent, and all protocols were approved by the Ethics Committee at the Tokyo Metropolitan Institute of Gerontology. We adhered strictly to the Declaration of Helsinki. A statement attached to the questionnaire explained the purpose of the survey and the voluntary nature of participation, and promised anonymity in the analysis.

### Definition of pet ownership

Participants were asked if they lived with a pet, i.e., dog, cat or other animals (current, past, or never). These responses were used to classify pet ownership as current, or past/never [1, 3, 16, 18].

### Medical and long-term care costs

In Japan, all citizens have access to medical care and long-term care under a universal health insurance system. The official medical insurance system comprises two categories. One is for employees and their dependents, and the other is the National Health Insurance (NHI) and health insurance for older people, including farmers, fishermen, and the self-employed, as well as retirees and pensioners, as beneficiaries. Japanese citizens are automatically enrolled in the health insurance for older people program on their 75th birthday. The NHI and health insurance for older people cover almost all medical treatment and medical provider fees [20]. Payments from insured persons to medical providers are made on a fee-for-service basis, in which the price of each service is determined by a uniform national fee schedule [20–23]. The Japanese LTCI system was established to support the need for long-term care services, community-based services, and in-facility services [24]. All primary insured persons aged 65 years or older are candidates for care, and secondary insured persons aged 40–64 years with any of 15 specific diseases can also utilize care services. Using data from the NHI, health insurance for older people, and LTCI beneficiaries in the town of Hatoyama, we calculated the monthly medical costs and monthly long-term care costs for each participant for a period of 17 months back from the baseline survey on June 2017, to assess the use of medical and long-term care services between pet owners and non-pet owners [20]. We used the 17-month period back from the survey to categorize participants as pet owners or not. Costs are expressed in Japanese yen or US dollars (1 US dollar = 115 Japanese yen in February 2022).

### Other measurements

We collected data on sociodemographic characteristics, including sex, age, living alone or co-habiting, self-reported long-term care level, history of chronic disease (hypertension, hyperlipidemia, heart disease, stroke, diabetes mellitus, bone or joint diseases, lung or respiratory illness, and cancer), alcohol drinking status, smoking status, exercise habits, frailty status, frequency of going outdoors, and self-rated health.

The chronic diseases that were evaluated included clinically relevant medical conditions. For each one, participants were asked if they had received a physician’s diagnosis (yes or no). Self-reported long-term care level was categorized as none, requiring help 1–2, long-term care level 1–2, or long-term care level 3–5 [25]. Frailty status was assessed by a modified version of the Kaigo-Yobo Checklist: scores ranged from 0 to 15, with a score higher than 4 defined as ‘frail’ [26]. Participants were asked about frequency of regular exercise: ≥5 times per week, 3–4 times per week, 1–2 times per week, 1–3 times per month, <1 time per month, or None.

### Statistical analyses

First, associations between baseline demographic and health characteristics, and pet ownership and non-ownership were tested using two-tailed Pearson’s chi-square or t-test. Second, pet ownership group-specific trajectories in the monthly medical and long-term care costs were modeled by a generalized estimating equation (GEE) with Poisson, respectively, including cubic terms for follow-up time and the first-order autoregressive correlation structure. GEE models can take into account correlation of within-subject data. Occurrence rates for medical and long-term care costs during the follow-up 18-month period were not statistically significant (χ^2^ P value = 0.622 and 0.958, respectively) between pet owners and non-owners, and we assigned medical and long-term care costs of 0 yen if participants did not use medical or long-term care services in any given month. To examine trajectories in the monthly medical and long-term care costs between pet owners and non-owners, we calculated monthly costs and accumulated costs for each participant for the 17 month period prior to the survey on June 2017. Assessment of the interaction between pet ownership and follow-up time with monthly costs was adjusted for sex, age, household size, and frailty status at baseline. Statistical analyses were conducted using SPSS (version 23.0; IBM Corp, Armonk, NY, USA) and SAS (version 9.4; SAS Institute, Inc., Cary, NC, USA). P values of less than .05 were considered statistically significant.

## Results and discussion

The mean (SD) age of participants was 77.7 (4.6) years, 61.6% of whom were men. Regarding help levels, 3.3% of participants self-reported a requirement for help level 1–2, while 1.3% self-reported a long-term care level of 1–2, 0.9% self-reported a long-term care level of 3–5, and 93.7% self-reported no long-term care. Slightly more than half (51.1%) had hypertension, 37.4% had hyperlipidemia, 27.4% had bone or joint disease, and 22.2% had heart disease. The percentage of participants who currently drank alcohol was 58.9%, while 6.3% currently smoked and 13.5% had frailty. Ninety-six participants (20.9%) were pet owners; the remaining 364 (79.1%) had no pet. Of the 96 pet owners, 24.0% owned a dog and a cat, 42.7% owned only a dog, 24.0% owned only a cat, and the remaining 9.4% owned another type of animal (Table 1). Except for frequency of regular exercise, pet owners and non-owners did not differ significantly in any baseline demographic or health characteristics.

**Table 1:** Baseline demographic and health characteristics among community-dwelling older Japanese pet owners and non-owners.

Variable	Pet owner (n = 96, 20.9%)	Non-pet owner (n = 364, 79.1%)	Total (n = 460)	P-Value
Sex (male %)	56.3	62.4	61.1	.275
Age (years)	77.1 (4.3)	77.9 (4.7)	77.7 (4.6)	.136
Household size (%)				.230
Living alone	9.4	14.0	22.2	
Living together	90.6	86.0	77.7	
Self-reported long-term care level				.266
None	92.7	94.0	93.7	
Requiring help 1–2	2.0	3.5	3.3	
Long-term care level 1–2	1.0	1.4	1.3	
Long-term care level 3–5	2.1	0.5	0.9	
Unknown or missing	2.1	0.6	0.9	
Chronic disease (%)				
Hypertension	47.9	51.9	51.1	.557
Hyperlipidemia	40.6	36.5	37.4	.352
Heart disease	26.0	21.2	22.2	.248
Stroke	9.4	11.0	10.7	.858
Diabetes mellitus	20.8	19.0	19.3	.538
Bone or joint diseases	25.0	28.0	27.4	.214
Lung or respiratory illness	14.6	13.2	13.5	.303
Cancer	14.6	14.0	14.1	.561
Alcohol drinking status (%)				.592
Current	60.4	58.5	58.9	
Past	8.3	9.6	9.3	
Never	31.3	31.9	31.7	
Smoking status (%)				.612
Current	5.2	6.6	6.3	
Past	43.8	38.5	39.6	
Never	51.0	54.9	54.1	
Frequency of regular exercise (%)				.041
≥5 times per week	49.0	32.2	35.7	
3–4 times per week	22.9	27.3	26.4	
1–2 times per week	14.6	23.1	21.4	
1–3 times per month	4.2	4.7	4.6	
<1 time per month	0	2.5	2.0	
None	9.4	10.2	10.0	
Frailty (%)	8.6	14.8	13.5	.119
Frequency of going outdoors (%)				.175
At least once a day	88.4	81.9	83.2	
Less than once every 2–3 days	11.6	17.8	16.6	
Missing	0	0.3	0.3	
Self-rated health (%)				.289
Excellent to good	81.3	85.4	84.6	
Fair to poor	17.7	14.0	14.8	
Missing	1.0	0.5	0.7	
Type of pet owned				
Dog and cat	24.0			
Dog	42.7			
Cat	24.0			
Other	9.4			

% or Mean (SD). P-values were calculated using a two-tailed Pearson’s chi-square test or T-test.

**Fig 1:**
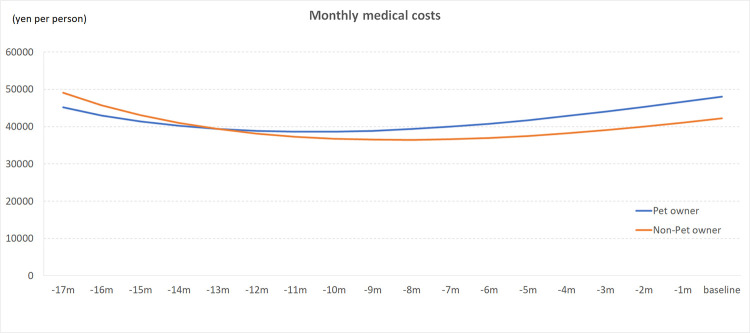
Trajectories of monthly medical costs by pet ownership group.

Fig 1 and Table 2 shows trajectories of the monthly medical costs in pet owners and non-owners. Pet owners and non-owners had estimated monthly medical costs of ¥48,054 (SE = 0.11; $418) and ¥42,260 (SE = 0.06; $367) at baseline, respectively. During the 18-month follow-up period, the non-pet owner to pet owner ratio of monthly medical costs was 0.9 at minimum and 1.2 at maximum, and accumulated medical costs for the 18 months were ¥753,140 ($6,549) in pet owners and ¥715,236 ($6,219) in non-owners. The association between pet ownership and follow-up time monthly medical costs was not significant (P = 0.68); pet owners and non-owners showed similar trends in the monthly medical costs.

**Table 2:** Monthly costs and accumulated costs for the 18-month period prior to the baseline survey.

		17m before	16m before	15m before	14m before	13m before	12m before	11m before	10m before	9m before	8m before	7m before	6m before	5m before	4m before	3m before	2m before	1m before	baseline
Pet owner																			
	estimated monthly medical costs (\)	45,157	43,035	41,414	40,218	39,388	38,876	38,645	38,666	38,912	39,363	39,999	40,802	41,754	42,838	44,034	45,318	46,668	48,054
	standard error	0.19	0.17	0.15	0.14	0.13	0.13	0.12	0.12	0.12	0.12	0.11	0.11	0.11	0.11	0.11	0.11	0.11	0.11
Non-pet owner																			
	estimated monthly medical costs (\)	49,078	45,716	43,054	40,969	39,364	38,166	37,317	36,771	36,493	36,453	36,626	36,992	37,533	38,232	39,071	40,035	41,105	42,260
	standard error	0.19	0.15	0.13	0.11	0.09	0.09	0.08	0.08	0.07	0.07	0.07	0.07	0.07	0.07	0.07	0.06	0.06	0.06
Pet owner																			
	estimated monthly care costs (\)	827	706	617	552	504	471	447	433	427	428	435	448	468	494	527	568	617	676
	standard error	0.67	0.65	0.65	0.66	0.68	0.70	0.72	0.74	0.75	0.77	0.78	0.79	0.79	0.79	0.78	0.78	0.77	0.75
Non-pet owner																			
	estimated monthly care costs (\)	1002	957	924	901	887	883	885	896	913	938	970	1,009	1,056	1,111	1,175	1,247	1,328	1420
	standard error	0.57	0.60	0.62	0.64	0.65	0.66	0.67	0.67	0.67	0.66	0.65	0.64	0.62	0.60	0.58	0.56	0.54	0.52

Estimated monthly medical and monthly care costs were Japanese yen. Monthly medical and long-term care costs were modeled by a generalized estimating equation with Poisson

**Fig 2:**
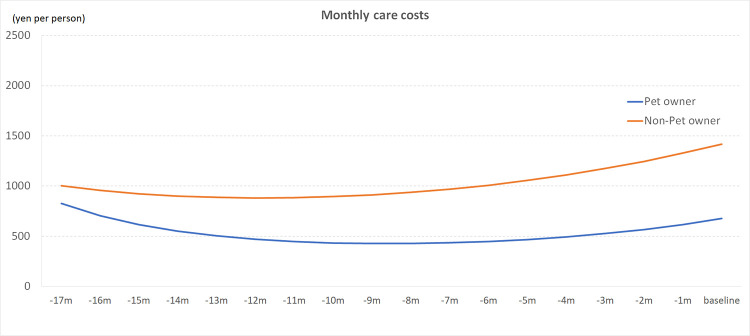
Trajectories of monthly long-term care costs by pet ownership group.

Trajectories of monthly long-term care costs in pet owners and non-owners are shown in Fig 2 and Table 2. Pet owners had estimated monthly long-term care costs of ¥676 (SE = 0.75; $6) at baseline, compared to ¥1,420 (SE = 0.52; $12) in non-owners. During the follow-up period, the non-pet owner to pet owner ratio for monthly long-term care costs was 1.2 at minimum and 2.3 at maximum. Accumulated long-term care costs were ¥9,645 ($84) in pet owners, but almost double this amount in non-owners, reaching ¥18,503 ($161). The interaction between pet ownership and follow-up time with monthly long-term care costs was significant (P = 0.03).

This study showed that monthly long-term care costs in elderly pet owners were approximately half of those in non-pet owners. Although we hypothesized that pet owners have both lower medical and long-term care costs than non-owners, only monthly long-term care costs showed lower estimated values. Interestingly, the long-term care costs of pet owners were reduced to approximately half of those of non-pet owners, despite no difference in self-reported long-term care levels. Daily pet care may be important for keeping regular hours, increasing physical activity, and facilitating social participation in older adults [1]. Physical and social advantages of pet ownership may underlie the reduced requirement for long-term care services and/or greater use of light care services, thereby contributing to lower monthly long-term care costs. A previous study reported that respondents who participated in social activities such as hobbies, group sports, or volunteering had lower cumulative cost of LTCI services over an 11-year period [27], which is consistent with our findings. In Japan, long-term care costs as a proportion of nominal GDP will increase by 4.7 percentage points by 2060, in the base case scenario [28]. The present study has shown that it is possible to reduce long-term care costs, which are expected to increase consistently, by promoting pet ownership among older adults.

Although monthly medical costs did not differ between pet owners and non-owners in the present study, the overall results show that use of medical services is strongly affected by factors other than physical, and support the psychological and social advantages of pet ownership. According to a survey from the Ministry of Health, Labour and Welfare in Japan [29], two major factors that affect increases in medical expenditure are aging and the sophistication of medical care (i.e., out-of-hospital care and internal medicine). In Japan, medical expenditure was about 42.2 trillion yen in 2020 and long-term care expenditure was about 10.7 trillion yen. How to curb increasing medical and long-term care costs is an urgent challenge.

We consider that the main strength of this study is that the data for calculating medical and long-term care costs were derived from the Japanese NHI, health insurance for older people, and LTCI beneficiaries. These systems cover nearly all medical provider fees and all care provider fees. Because Japan has a universal health insurance system, we were able to link pet ownership and medical and long-term care costs for community-dwelling older people.

Among the study’s limitations are, first, the sample size of 460 participants precluded analysis of specific types of pets. This study defined pets as dog, cat or other animals, however detailed information for other animals is not clear. Previous studies have reported superior protective effects against adverse health outcomes for dog ownership compared to cat ownership [16–18]. Further research is needed to examine the medical and long-term care costs among dog owners compared to cat owners. Second, due to small sample size with limited statistical power in this study, only a few confounding factors were selected to examine the interaction between pet ownership and follow-up period, including sex, age, household size, and frailty status. Also, the data on self-rated economic status was collected in 2016. This measure did not differ significantly between pet owners and non-owners, and the adjusted model with gender, age, and self-rated economic status did not influence the interaction of pet ownership and follow-up time with monthly medical and long-term care costs. Future studies with larger sample sizes and statistical matching are necessary to examine the medical and long-term care costs among pet owners and non-owners after adjusting for various potential confounders. Third, we used 17 months back from the survey to establish pet ownership; however, we did not collect data on the number of years of pet ownership. Future study should assess the history of pet ownership in more detail.

## Conclusions

This study is the first to show that monthly long-term care costs in pet owners were approximately half of those in non-pet owners. We raise the possibility of reducing long-term care costs by promoting pet ownership among older adults, which could have important implications for sustaining the social security system.
